# Sex-Based Differences in Treatment with Immune Checkpoint Inhibition and Targeted Therapy for Advanced Melanoma: A Nationwide Cohort Study

**DOI:** 10.3390/cancers13184639

**Published:** 2021-09-16

**Authors:** Monique K. van der Kooij, Olaf M. Dekkers, Maureen J. B. Aarts, Franchette W. P. J. van den Berkmortel, Marye J. Boers-Sonderen, Jan Willem B. de Groot, Geke A. P. Hospers, Djura Piersma, Rozemarijn S. van Rijn, Karijn P. M. Suijkerbuijk, Hans M. Westgeest, Astrid A. M. van der Veldt, Gerard Vreugdenhil, Sofie Wilgenhof, Michel W. J. M. Wouters, John B. A. G. Haanen, Alfonsus J. M. van den Eertwegh, Ellen Kapiteijn

**Affiliations:** 1 Department of Medical Oncology, Leiden University Medical Center, Albinusdreef 2, P.O. Box 9600, 2300 RC Leiden, The Netherlands; m.k.van_der_kooij@lumc.nl; 2 Department of Clinical Epidemiology, Leiden University Medical Center, Albinusdreef 2, P.O. Box 9600, 2300 RC Leiden, The Netherlands; o.m.Dekkers@lumc.nl; 3 Department of Medical Oncology, Maastricht University Medical Center, P. Debyelaan 25, 6202 AZ Maastricht, The Netherlands; mjb.essers.aarts@mumc.nl; 4 Department of Medical Oncology, Zuyderland Medical Center, 6130 MB Sittard-Geleen, The Netherlands; f.vandenberkmortel@zuyderland.nl; 5 Department of Medical Oncology, Radboud University Medical Center, Geert Grooteplein Zuid 10, 6500 HB Nijmegen, The Netherlands; Marye.Boers-Sonderen@radboudumc.nl; 6 Isala Oncology Center, Isala, 8000 GK Zwolle, The Netherlands; j.w.b.de.groot@isala.nl; 7 Department of Medical Oncology, University Medical Center Groningen, Hanzeplein 1, 9713 GZ Groningen, The Netherlands; g.a.p.hospers@umcg.nl; 8 Department of Medical Oncology, Medisch Spectrum Twente, Koningsplein 1, 7512 KZ Enschede, The Netherlands; D.Piersma@mst.nl; 9 Department of Medical Oncology, Medical Center Leeuwarden, Henri Dunantweg 2, 8934 AD Leeuwarden, The Netherlands; Rozemarijn.van.Rijn@ZNB.NL; 10 Department of Medical Oncology, University Medical Center Utrecht, Heidelberglaan 100, 3584 CX Utrecht, The Netherlands; K.Suijkerbuijk@umcutrecht.nl; 11 Department of Internal Medicine, Amphia Ziekenhuis, Molengracht 21, 4818 CK Breda, The Netherlands; HWestgeest@amphia.nl; 12 Departments of Medical Oncology and Radiology & Nuclear Medicine, Erasmus MC Cancer Institute, Dr. Molewaterplein 40, 3000 CA Rotterdam, The Netherlands; a.vanderveldt@erasmusmc.nl; 13 Department of Medical Oncology, Maxima Medical Center, de Run 4600, 5500 MB Veldhoven, The Netherlands; G.Vreugdenhil@mmc.nl; 14 Department of Medical Oncology, Netherlands Cancer Institute—Antoni van Leeuwenhoek Hospital, Plesmanlaan 121, 1066 CX Amsterdam, The Netherlands; s.wilgenhof@nki.nl (S.W.); j.haanen@nki.nl (J.B.A.G.H.); 15 Dutch Institute for Clinical Auditing, Rijnsburgerweg 10, 2333 AA Leiden, The Netherlands; m.wouters@nki.nl; 16 Department of Surgical Oncology, Netherlands Cancer Institute—Antoni van Leeuwenhoek Hospital, Plesmanlaan 121, 1066 CX Amsterdam, The Netherlands; 17 Department of Medical Oncology, Cancer Center Amsterdam, Amsterdam UMC, Vrije Universiteit Amsterdam, de Boelelaan 1117, 1081 HZ Amsterdam, The Netherlands; vandeneertwegh@vumc.nl

**Keywords:** sex, advanced melanoma, immunotherapy, targeted therapy, prospective nation-wide data

## Abstract

**Simple Summary:**

Melanoma is a malignant form of skin cancer. The overall survival of patients with advanced stages of disease were initially low. Fortunately, in recent years systemic treatment with immunotherapy has prolonged survival. We set out to answer the question whether men and women with advanced melanoma differ in prognostic factors, tumor-response to immunotherapy, and treatment-related adverse events. All patients in the Netherlands were registered between July 2013 and July 2018. We showed that although clinical and tumor characteristics differ, the safety profile of immunotherapy is comparable. Furthermore, overall, a 10% survival advantage for women was seen. Following immunotherapy there was no survival difference.

**Abstract:**

Recent meta-analyses show conflicting data on sex-dependent benefit following systemic treatment for advanced melanoma patients. We examined the nationwide Dutch Melanoma Treatment Registry (July 2013–July 2018), assessing sex-dependent differences in advanced melanoma patients (stage IIIC/IV) with respect to clinical characteristics, mutational profiles, treatments initiated, grade 3–4 adverse events (AEs), treatment responses, and mortality. We included 3985 patients, 2363 men (59%) and showed that although men and women with advanced melanoma differ in clinical and tumor characteristics, the safety profile of immune checkpoint inhibition (ICI) is comparable. The data suggest a 10% survival advantage for women, mainly seen in patients ≥60 years of age and patients with BRAF V600 mutant melanoma. Following ICI there was no survival difference.

## 1. Introduction

Immunotherapy is currently changing the landscape of oncology. Systemic treatment with immune checkpoint inhibition (ICI) targeting programmed cell death 1 (anti-PD-1) and cytotoxic T-lymphocyte antigen-4 (anti-CTLA-4) can overcome tumor-induced immunosuppression in advanced malignancies [1]. BRAF, NRAS and c-KIT mutations in melanoma have shown to be distinct clinic-pathological entities [2]. Targeted therapy with BRAF-inhibition has demonstrated clear antitumor activity in patients whose tumors harbor the characteristic BRAF V600E or V600K mutation [3,4]. The addition of a MEK-inhibitor has shown to lead to more (durable) clinical responses [5]. Interestingly, membrane-bound estrogen receptors were shown to be responsible for an increased activity of the RAS/BRAF/MEK axis [6].

Components of both the innate and the adaptive immune system are differently regulated in men and women. Female patients have a faster clearance of pathogens and greater vaccine efficacy, but are more prone to inflammatory and autoimmune diseases. Contrarily, men have an almost twofold greater risk of mortality from malignant cancers [7].

In oncologic patients, it was recently shown that women are prone to stronger immunoediting in early tumor development. ICI in a later stage could therefore have a reduced effect in women, as this treatment will reactivate T cells for immunologically invisible (neo)antigens [8]. Furthermore, several studies reported differences between men and women in (possible) biomarkers for the response to ICI, including; tumor mutational burden, neoantigen load, PD-L1 expression, DNA mismatch repair deficiency, cytotoxic T cell infiltration, gene-expression and mutational signatures, antigen presentation defects, sex hormones, and interferon signaling [9,10,11,12,13,14,15,16,17,18,19,20].

In recent years, studies investigating the sex-dependent magnitude of benefit following treatment with ICI showed contradicting results. The first study showed that men derived greater value from ICI as compared to women [21]. Two more recent meta-analyses included several comprehensive and updated studies. These analyses concluded that there was no clear association between sex and the efficacy of ICI in the treatment of advanced cancers, including melanoma [22,23]. A fourth meta-analysis focused on anti-PD-1/anti-PD-L1 treatment in patients with advanced and metastatic cancer, including melanoma. They also could not show an overall survival (OS) difference between male and female patients [24].

The previously mentioned meta-analyses included large randomized controlled trials, however, a vast proportion of patients with advanced melanoma treated in daily practice do not meet the in- and exclusion criteria of these trials [25,26]. Another limitation of these analyses was that the authors lacked additional information on patient-specific data, including the distribution of known risk factors among men and women [27]; this is important as the comparison between men and women in the setting of a randomized controlled trial can still be confounded, as it is not sex that is randomized. Potential differences in these prognostic markers, and tumor response following treatment between male and female patients could indicate that sex should be taken into account in the assessment of risk versus benefit when making decisions about treatment strategies.

Therefore, using our population-based cohort of unresectable stage IIIC and IV melanoma patients, we set out to answer the question whether men and women differ in baseline and tumor characteristics, first-line systemic treatments initiated and the safety and efficacy of targeted therapy and ICI.

## 2. Materials and Methods

### 2.1. Dutch Melanoma Treatment Registry

Since 2013, all advanced melanoma patients in the Netherlands are referred to one of 14 expert hospitals and data are prospectively registered in the DMTR (Dutch Melanoma Treatment Registry). To assure safety and quality of melanoma care in the Netherlands centralization of advanced melanoma patients and subsequent their registration in the DMTR was initiated [28]. Information on patients’ baseline and tumor characteristics, treatment regimens, grade 3–4 treatment related AEs (according to the Common Terminology Criteria for Adverse Events, version 4.0), clinical outcomes and date of death are registered. These data are collected from patient files by trained data managers and approved by the treating physicians. The DMTR was approved by a medical ethical committee (METC Leiden University Medical Center, Leiden, The Netherlands, 2013) and is not considered subject to the Medical Research Involving Human Subjects Act.

### 2.2. Patients, Treatments and Outcome Definitions

Data on all patients diagnosed with unresectable stage III or IV melanoma in the Netherlands between July 2013 and July 2018 were retrieved, follow-up data cut-off was set at 1 March 2019. The patient with missing data on gender (*N* = 1) was excluded from the analysis. After describing the location of primary tumor in male and female patients, patients with mucosal and uveal melanoma were excluded (*N* = 375). Patients with a melanoma of unknown primary were included in the analyses.

First-line anti-cancer systemic treatment strategies were compared between men and women, and included: chemotherapy with dacarbazine, ICI with anti-CTLA-4 (ipilimumab), anti-PD-1 (nivolumab, or pembrolizumab), or combination treatment with anti-CTLA-4 and anti-PD-1 (nivolumab and ipilimumab), targeted therapy with BRAF-inhibitors (vemurafenib, dabrafenib, encorafenib) and MEK-inhibitors (trametinib, cobimetinib, binimetinib), or “other”. Safety analysis was based on comparison of grade 3–4 AEs, and death due to adverse events (grade 5). Clinical outcomes were collected for all patients. The best overall response (BOR) is the best evaluation that a patient received after initiation of treatment, until the start of new melanoma therapy, or the last follow-up visit; progressive disease (PD), stable disease (SD), partial response (PR), or complete response (CR). The overall response rate (ORR) is defined as the proportion of patients who have a PR or CR following therapy. Survival time for all patients was calculated from the date of diagnosis of advanced melanoma to the date of the last follow-up visit (censored observation) or date of death as a result of any cause.

### 2.3. Statistical Analysis

Continuous variables were compared using a *t*-test, and chi-squared tests for categorical variables. All statistical tests were two-sided, and a *p* value of less than 0.05 was considered statistically significant. Potential differences between treatment choices in men and women after correcting for the presence of a BRAF V600 mutation were analyzed.

Progression free survival (PFS), overall survival (OS) and disease specific survival (DSS) were used as measure of survival probabilities. The cumulative incidence competing risk method was used to estimate melanoma-related mortality risk. To estimate sub-distribution Hazard Ratio (sHR) and corresponding 95% CIs, Fine and Gray competing risk models were used with melanoma-related death as event and non-melanoma related death as competing risk. Risk factors that were included in the Cox proportional hazard and competing risk models were: age, ECOG performance status (0, 1, or ≥2), LDH level (not elevated, elevated within 2× upper limit of normal, or strongly elevated >2× upper limit of normal), presence of brain metastases, presence of distant metastasis in ≥3 organ sites, and *BRAF mutation* (presence of targetable—BRAFV600E or BRAFV600K—mutation). Patients that received BRAF inhibition were assumed to have a targetable BRAFV600 mutation in their tumor. Additionally, patients were stratified in age-groups corresponding with presumed hormonal status; pre-menopausal (≤45), menopausal (46–59) and post-menopausal (≥60 years of age). The peri-menopausal status was defined around the mean age of menopause, which is 50–51 years in Western countries and is in accordance with previously published research [29,30,31]. The proportional hazards assumption was checked by visual inspection.

Crude HRs and adjusted HRs for the above-mentioned risk factors and treatment groups were estimated. To test whether sex HRs differed across subgroups, an interaction term between sex and the subgroup variable was used.

SPSS version 25.0 (IBM Corp. Released 2017. IBM SPSS Statistics for Windows, Version 25.0, Armonk, NY, USA, IBM Corp) was used to perform the descriptive statistics, Cox regression, Pearson Chi-Square analysis and survival analysis according to the Kaplan-Meier’s method to calculate risk estimates. STATA version 14.1 (StataCorp. 2015. Stata Statistical Software: Release 14. College Station, TX, USA, StataCorp LP.) was used to calculate cumulative incidence function in the presence of the competing risk (non-melanoma related death). Figures were created in GraphPad Prism version 8.1.1 (GraphPad Software, La Jolla, CA, USA).

## 3. Results

### 3.1. Baseline Characteristics

**Figure 1:**
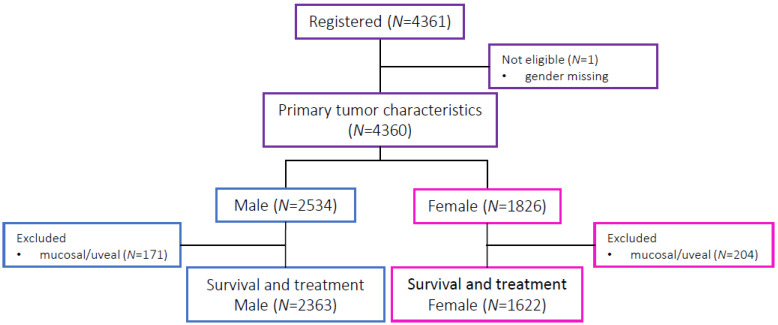
Patient selection for statistical analysis.

4361 advanced melanoma patients were registered; after excluding patients with mucosal and uveal melanoma, 3985 patients were selected; 2363 men (59.3%) and 1622 (40.7%) women, see Figure 1.

Clinical characteristics at time of advanced disease are shown in Table 1. Women were younger, with a median age of 63 versus 65 years (*p* < 0.001), had a lower M-stage (AJCC v7) at time of diagnosis (*p* = 0.001), and less often showed metastases in > 3 organ sites (29.9 versus 34.8%, *p* = 0.001).

The anatomical location and clinical characteristics of the primary tumor are shown in Figure S1. In men the primary tumor was more often located in the head/neck and trunk (16 versus 9%), while in women it was more frequently located on the extremities (21 versus 36%). The primary melanomas of male patients were thicker, with more ulceration and were more frequently nodular. Female patients had a longer time gap between primary disease and development of advanced disease (58 versus 43 months).

### 3.2. Tumor Mutational Status

Overall, mutational pattern of the tumor differed between men and women, *p* < 0.001. Female patients more frequently harbored BRAF V600E mutant melanoma (46% versus 36%), while BRAF V600K and NRAS mutations were more prevalent in the tumors of male patients (8% versus 4% and 21% versus 18%, respectively). There was an age-dependent decrease in BRAF V600 mutations, while the percentage of patients harboring an NRAS mutation increased. In all age-groups BRAF V600E mutations were more frequently found in the tumors of female patients, whereas male patients more often carried a BRAF V600K or NRAS mutation, see Figure S2.

### 3.3. Initial Systemic Treatment Initiated

**Figure 2:**
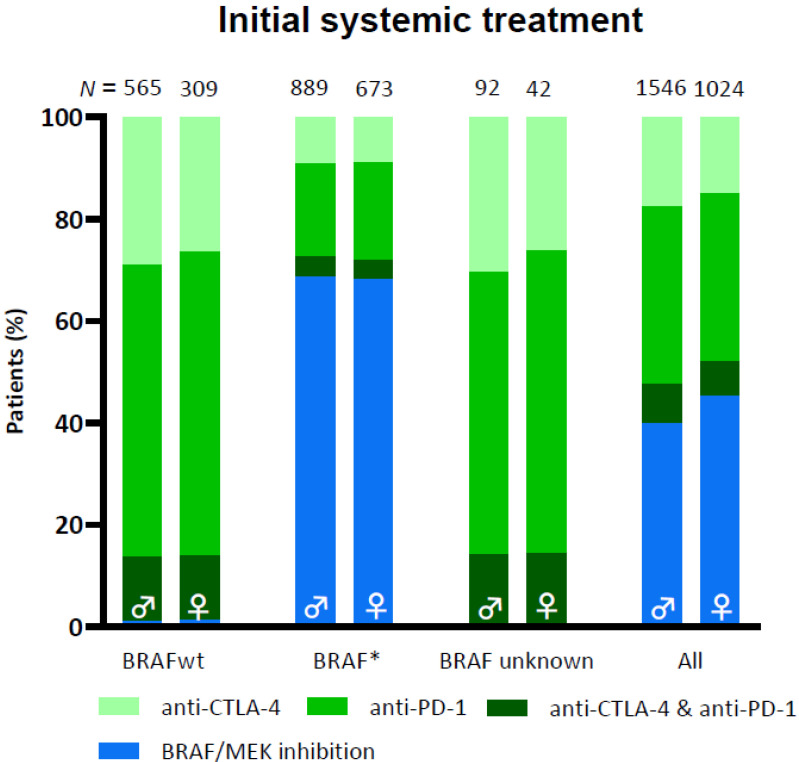
Initial systemic treatment with immune checkpoint inhibition and targeted therapy in male and female patients. “*”: mutation.

In 1736 men (74%) and 1180 women (73%) systemic therapy was the first-line treatment. Male patients more frequently received ICI (40% versus 35%), while targeted therapy was given more frequently to female patients (29% versus 26%). This difference was related to the presence of a BRAF mutation and disappeared after stratification; BRAF wild type (*p* = 0.26), BRAF V600 mutant (*p* = 0.90), and no BRAF mutational status determined (*p* = 0.54), see Figure 2.

### 3.4. Treatment Safety

#### 3.4.1. Targeted Therapy (BRAF/MEK Inhibition)

Treatment with targeted therapy gave more grade 3–4 AEs in women, 25% versus 20%, respectively (*p* = 0.06) (Table 2). No clear difference in the type of AEs (Table 2) was found.

#### 3.4.2. Immune Checkpoint Inhibition (Anti-CTLA-4, Anti-PD-1 and the Combination)

ICI with anti-CTLA-4 and anti-PD-1 resulted in similar percentages of AEs in men and women, which remained after adjusting for age. Furthermore, there was no difference in the type of AEs between these groups (Table 2). Adjustment for age made no material difference.

### 3.5. Treatment Efficacy

Response rates (ORR; PR or CR) following ICI with either anti-CTLA-4 (20 versus 18%, *p* = 0.62) or anti-PD-1 (53 versus 51%, *p* = 0.59) were similar for men and women. However, men had lower ORRs compared to women following targeted therapy (52 versus 58%, *p* = 0.07) and combination treatment with anti-CTLA-4 + anti-PD-1 (51 versus 67%, *p* = 0.06), see Table S1. This difference in response remained after adjusting for the previously described prognostic factors, see Table S1.

### 3.6. Survival

Median OS was 59 weeks in male patients and 71 weeks in female patients. After adjusting for prognostic factors, adjHRs for women when compared to men were 0.92 (95% CI 0.84–0.99) for OS, 0.89 (95% CI 0.81–0.98) for DSS (0.92 (95% CI 0.83–1.01) when accounting for the competing risks) (Table 3).

Following targeted therapy, female patients had a longer PFS (adjHR 0.85, 95% CI 0.73–0.99) and a better OS (adjHR of 0.89, 95% CI 0.77–1.03) compared to male patients. There was no difference in survival following ICI monotherapy with; anti-CTLA-4, adjHR 0.86 (95% CI 0.66–1.10) or anti-PD-1, adjHR 1.11 (95% CI 0.89–1.38). Although the number of patients treated with combination therapy anti-CTLA-4 + anti-PD-1 was limited (*n* = 190), the point estimate suggests a possible survival advantage for women when compared to men HR 0.66 (95% CI 0.38–1.13).

When stratifying all patients across menopausal age categories, differences in adjusted HRs for OS and DSS were mainly seen in patients ≥60 years of age (Table S2). Furthermore, survival advantage of female patients treated with targeted therapy was also mainly seen in the postmenopausal age group with adjusted HRs for PFS 0.72 (95% CI 0.58–0.89), OS 0.69 (95% CI 0.57–0.85) and DSS 0.75 (95% CI 0.59–0.94). In the younger age groups, there were not enough patients treated with ICI to reliably estimate adjHRs (Table S2).

#### BRAF V600 Mutation

**Figure 3:**
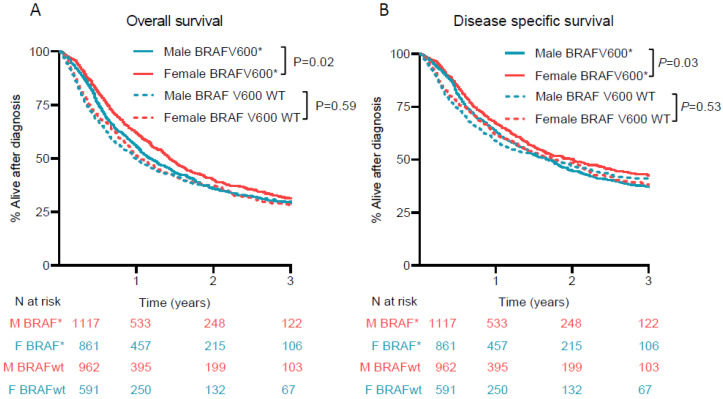
Overall and disease specific survival in men and women stratified by BRAF mutational status (**A**) Overall survival in years since diagnosis of advanced melanoma in patients with a BRAF V600 mutation (BRAFV600 */BRAF *) and patients proven to be BRAF V600 wild type (BRAF V600 WT/BRAFwt). (**B**) Disease specific survival in years is shown since diagnosis of advanced melanoma in patients with a BRAF V600 * and patients proven to be BRAF V600 WT. M = men, W = women, “*” = mutation.

OS advantage of women could only be observed in patients harboring a BRAF V600 mutation, adjHR 0.87 (95% CI 0.78–0.98) and remained after restriction to BRAF V600E mutations. The same pattern could be observed for DSS, see Figure 3.

### 3.7. Risk Factors for Overall Survival in Male and Female Patients

**Figure 4:**
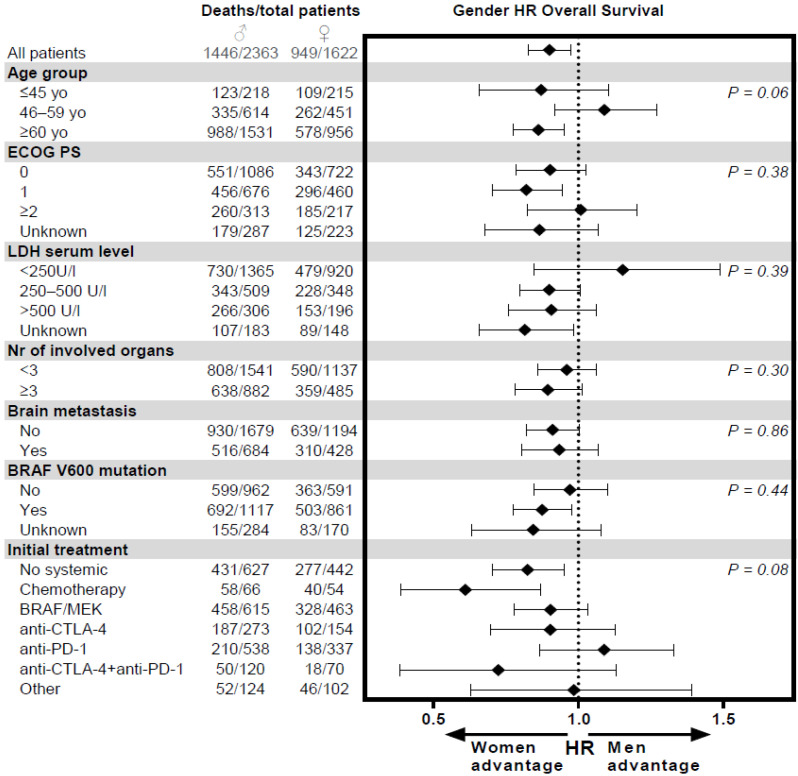
Subgroup analyses for overall survival. Subgroup analyses presented show crude sex HRs for overall survival. *p*-values presented show the statistical significance of the interaction term of the presented prognostic factor and sex in a Cox proportional hazard model.

Forest plots of the subgroup analyses of the sex difference for OS are shown in Figure 4, including *p*-values for interaction of these subgroups with sex. The female patient survival advantage was observed in the majority of subgroups, including the subgroup of female patients that was not systemically treated. Women seemed to have equal advantage with high or low tumor-burden; the HR remained similar in patients with <3 versus ≥3 organs involved and showed only a slight decrease in patients with a higher LDH serum level.

## 4. Discussion

The data from this nation-wide study show that female patients with advanced melanoma have an OS advantage of approximately 10% over male patients. However, this difference appeared to be driven by the subgroups of postmenopausal women and female patients with a BRAF V600 mutant melanoma.

From previous research it is known that men, compared to women, are less likely to self-detect their melanomas [33] and make fewer visits to healthcare providers [34]. This could result in diagnostic delay in men, explaining the baseline differences found in our study. Corresponding with a diagnosis at an earlier time, female patients had thinner primary melanomas, less ulceration, and less nodular melanomas. Once women developed advanced melanoma they had a lower M-stage with less organ sites affected by distant metastases. However, the time-gap between primary and advanced melanoma was longer in female patients. This indicates a less aggressive tumor proliferation in female patients or a stronger anti-tumor response in early tumor development [8].

Historically, the presence of a BRAF V600 mutation was associated with more aggressive tumor features and a shorter survival [35,36]. Due to the introduction of BRAF- and MEK-inhibition, this mutation has become a target for anti-tumor treatment. Our data show that advanced melanoma in women more frequently harbors a BRAF V600E mutation, while melanoma in men more frequently has a NRAS or BRAF V600K mutation. Our data strengthen data from previously published smaller cohorts [37,38,39].

The increased ratio of BRAF mutant melanomas in female versus male patients resulted in more targeted therapy initially being prescribed to female patients. Although this treatment did lead to more grade 3–4 AEs, it also yielded a higher ORR in women, which translated into a longer PFS.

The safety profiles of ICI were similar in men and women. Data on our 427 patients treated with anti-CTLA-4 contradicts previously published data on 140 patients by Valpione et al. [40], who reported that more AEs occurred in female patients.

Multiple retrospective and some prospective trials and meta-analyses have investigated sex as a prognostic factor for survival in (advanced) melanoma. Possible explanations for sex differences were: age at diagnosis, disease severity, tumor composition and infiltration, influence of estrogens in female patients, and overall longevity of women. Our current findings show that the survival advantage is mainly seen in the older (postmenopausal) age-group which supports the hypothesis that this might be due to female longevity. On the contrary, the observation that there was no difference in the efficacy of ICI over the different age-groups contradicts the influence of estrogens in female patients.

Before the introduction of ICI and targeted therapy, a pooled analysis of five EORTC randomized trials with metastatic melanoma showed that women had a better OS, DSS and PFS when compared to men. This difference decreased in female patients with more advanced disease [31]. These results were similar to a paper on the American SEER database, including melanoma patients with localized, regional, and metastatic disease [41]. Our study reports a female OS advantage in both patients with more and less advanced disease, in the era of ICI and targeted therapy.

A major strength of our population-based registry over the meta-analyses discussed in the introduction is that we also report data from patients with more advanced melanoma and a worse clinical performance score that do not meet the in- and exclusion criteria [25,26]. Another advantage of our registry is that we were able to adjust survival for patient baseline (tumor) characteristics and known risk factors. Furthermore, the data shown is from a more homogeneous group when compared to some meta-analyses that include patients irrespective of tumor type.

A limitation of our study is that data on hormonal status groups was based on age. Furthermore, not all patients progressed on their initial treatment before the start of a second line of systemic therapy. For example, treatment with targeted therapy could be given as an induction therapy. Therefore, data on ORR and PFS will be less reliable when compared to OS. The number of patients treated with combination treatment anti-CTLA-4 and anti-PD-1 was limited, therefore results on toxicity and efficacy of this treatment regimen have to be interpreted with caution. Additionally, as all patients in the Netherlands were included, systemic therapy could have been given as part of a clinical trial.

## 5. Conclusions

Our study shows that female advanced melanoma patients have an OS advantage of approximately 10% over male patients. Furthermore, women treated with targeted therapy have a better ORR and PFS, leading to a better OS in women with a BRAF V600 mutant melanoma over men. This difference was not seen in the patients without this mutation, nor in male and female patients initially treated with ICI. The usage of a population-based registry with national coverage omits limitations from large phase III trials by also including patients that would not be eligible for studies. We encourage the use of this population-based data in the future to compare treatment choices, and to complement information that is provided by meta-analyses on drug safety and efficacy.

## Figures and Tables

**Table 1:** Clinical and tumor characteristics of advanced cutaneous melanoma patients.

Characteristics at Baseline	Men *N* = 2363 (%)		Women *N* = 1622 (%)		*p* Value
Time since primary (months)	43	(0–841)	58	(0–603)	<0.001
Median age, year (range)	65	(15–97)	63	(17–96)	<0.001
Age categories					<0.001
≤45 years	218	(9.2%)	215	(13.3%)	
46–59 years	614	(26.0%)	451	(27.8%)	
≥60 years	1531	(64.8%)	956	(58.9%)	
ECOG PS					0.49
0	1086	(46.0%)	722	(44.5%)	
1	676	(28.6%)	460	(28.4%)	
≥2	313	(13.3%)	217	(13.4%)	
Unknown	287	(12.2%)	223	(13.7%)	
LDH					0.42
Normal (<250 U/L)	1365	(57.8%)	930	(57.3%)	
250–500 U/L	509	(21.5%)	348	(21.5%)	
>500 U/L	306	(12.9%)	196	(12.1%)	
Unknown	183	(7.7%)	148	(9.1%)	
M-stage					0.001
M1a	248	(10.5%)	218	(13.4%)	
M1b	263	(11.1%)	155	(9.6%)	
M1c	1804	(76.3%)	1194	(73.6%)	
Unknown	48	(2.0%)	55	(3.4%)	
Metastasis in ≥ 3 organ sites	822	(34.8%)	485	(29.9%)	0.001
Brain metastasis					
Yes	684	(28.9%)	428	(26.4%)	0.08
Symptomatic	487	(71.2%)	270	(63.1%)	0.005
Asymptomatic	197	(28.8%)	158	(36.9%)	
BRAF mutation					
V600 *	1117	(47.3%)	861	(53.1%)	0.001
V600E	866	(36.6%)	748	(46.1%)	
V600K	191	(8.1%)	71	(4.4%)	

ECOG PS: Eastern Cooperative Oncology Group Performance Status [32], LDH: Lactate dehydrogenase, M-stage: location of distant metastasis (M1a: skin and/or soft-tissue, M1b: lung, M1c: any other location), “*”: mutation.

**Table 2:** Adverse events following systemic therap.

Adverse Events	Men *N* (%)	Women *N* (%)	*p* Value
BRAF/MEK inhibition	614	463	
Grade 3–4	124 (20.2)	115 (25.1)	0.06
Skin/eye	56 (45.2)	55 (47.8)	
GI/Liver	41 (33.1)	38 (33.0)	
Other	54 (43.5)	39 (33.9)	
Grade 5	0	1 (0.2)	
Anti-CTLA-4	273	154	
Grade 3–4	87 (31.9)	49 (31.8)	0.99
GI/Liver	52 (59.8)	29 (59.2)	
Endocrine	20 (23.0)	12 (24.5)	
Skin	10 (11.5)	2 (4.2)	
Myelotoxicity	4 (4.6)	0	
Neurological/Uveitis	1 (1.1)	1 (2.0)	
Other	16 (18.4)	7 (14.6)	
Grade 5	2 (0.7)	0	
Anti-PD-1	513	324	
Grade 3–4	75 (14.6)	42 (13.0)	0.50
GI/Liver	24 (32.0)	16 (38.1)	
Endocrine	8 (10.7)	2 (4.8)	
Skin	5 (6.7)	6 (14.3)	
Renal	7 (9.3)	3 (7.1)	
Respiratory	9 (12.0)	4 (9.5)	
Myelotoxicity	2 (2.7)	0	
Neurological/Uveitis	2 (2.7)	0	
Other	30 (40.0)	19 (45.2)	
Grade 5	1 (1.4)	2 (4.9)	
Anti-CTLA-4 + anti-PD-1	120	70	
Grade 3–4	68 (56.7)	40 (57.1)	0.95
GI/Liver	48 (70.6)	28 (70.0)	
Endocrine	11 (16.2)	9 (22.5)	
Skin	7 (10.3)	4 (10.0)	
Renal	3 (4.5)	1 (2.5)	
Respiratory	4 (6.1)	4 (10.0)	
Myelotoxicity	1 (1.5)	0	
Neurological	2 (2.9)	2 (5.0)	
Other	13 (19.1)	10 (25.0)	
Grade 5	1 (0.8)	0	

**Table 3:** Survival of female compared to male patients following initial systemic treatments.

Treatment Groups		Events/Total (*N*)			
		Men	Women	HR (95% CI)	adjHR (95% CI)
All patients	OS	1446/2363	949/1622	0.90 (0.83–0.98)	0.92 (0.84–0.99)
	DSS	1109/2363	709/1622	0.88 (0.80–0.96)	0.89 (0.81–0.98)
	Comp. risk	1109/2363	709/1622	0.90 (0.82–0.98)	0.92 (0.83–1.01)
Initial treatment					
BRAF/MEK inhibition	OS	457/614	328/463	0.90 (0.78–1.04)	0.89 (0.77–1.03)
	DSS	375/614	259/463	0.87 (0.74–1.02)	0.86 (0.73–1.01)
	Comp. risk	375/614	259/463	0.89 (0.76–1.05)	0.90 (0.76–1.06)
	PFS	416/614	292/463	0.86 (0.74–1.00)	0.85 (0.73–0.99)
Anti-CTLA-4	OS	187/273	102/154	0.89 (0.70–1.13)	0.86 (0.66–1.10)
	DSS	153/273	83/154	0.88 (0.67–1.15)	0.84 (0.64–1.11)
	Comp. risk	153/273	83/154	0.88 (0.68–1.15)	0.84 (0.63–1.12)
	PFS	247/273	140/154	0.96 (0.78–1.19)	0.95 (0.77–1.18)
Anti-PD-1	OS	210/536	138/336	1.07 (0.86–1.32)	1.11 (0.89–1.38)
	DSS	156/536	106/336	1.10 (0.86–1.41)	1.13 (0.88–1.46)
	Comp. risk	156/536	106/336	1.10 (0.86–1.41)	1.14 (0.87–1.49)
	PFS	333/536	211/336	1.07 (0.90–1.27)	1.07 (0.90–1.28)
Anti-CTLA-4 + anti-PD-1	OS	50/120	18/70	0.66 (0.38–1.13)	—
	DSS	47/120	15/70	0.58 (0.32–1.04)	—
	Comp. risk	47/120	15/70	0.58 (0.32–1.04)	—
	PFS	77/120	32/70	0.74 (0.48–1.12)	—

Events and total number of men and women is shown, followed by hazard ratio and corresponding 95% confidence interval, and the adjusted hazard (adjHR) ratio with 95% confidence interval for overall survival (OS), disease specific survival (DSS), and progression free survival (PFS). Hazard ratios were adjusted for: sex, age, ECOG performance status, LDH, ≥3 organ sites affected, the presence of brain metastases, and BRAF V600 mutation status. Only for patients treated with targeted therapy was the BRAF V600 mutational status not included in the Cox proportional hazard model. Due to the limited number of patients treated with combination therapy with anti-CTLA-4 and anti-PD-1, no adjHRs were calculated for this subgroup of patients.

## Data Availability

Study protocol: Available from M.K.v.d.K. (e-mail, m.k.van_der_kooij@lumc.nl). Statistical code: Not available. Data set: Can be applied for at https://dica.nl/dmtr/onderzoek (accessed on 1 March 2019).

## Supplementary Materials

The following are available online at https://www.mdpi.com/article/10.3390/cancers13184639/s1, Figure S1: Characteristics of primary tumors of men and women, Figure S2: Mutational pattern of the tumor in men and women with advanced melanoma, stratified by age-groups, Table S1: Best overall response rate following systemic therapy, Table S2: Survival in different age categories.
