# Supplementary material for: Sex-Based Differences in Treatment with Immune Checkpoint Inhibition and Targeted Therapy for Advanced Melanoma: A Nationwide Cohort Study
Source: Cancers (Basel). 2021 Sep 16;13(18):4639. doi: 10.3390/cancers13184639 (PMC8465427; doi:10.3390/cancers13184639)
Supplement: Supplementary file 1 [file cancers-13-04639-s001.zip › cancers-1367334-supplementary.pdf]

cancers

MDPI

Supplementary Materials

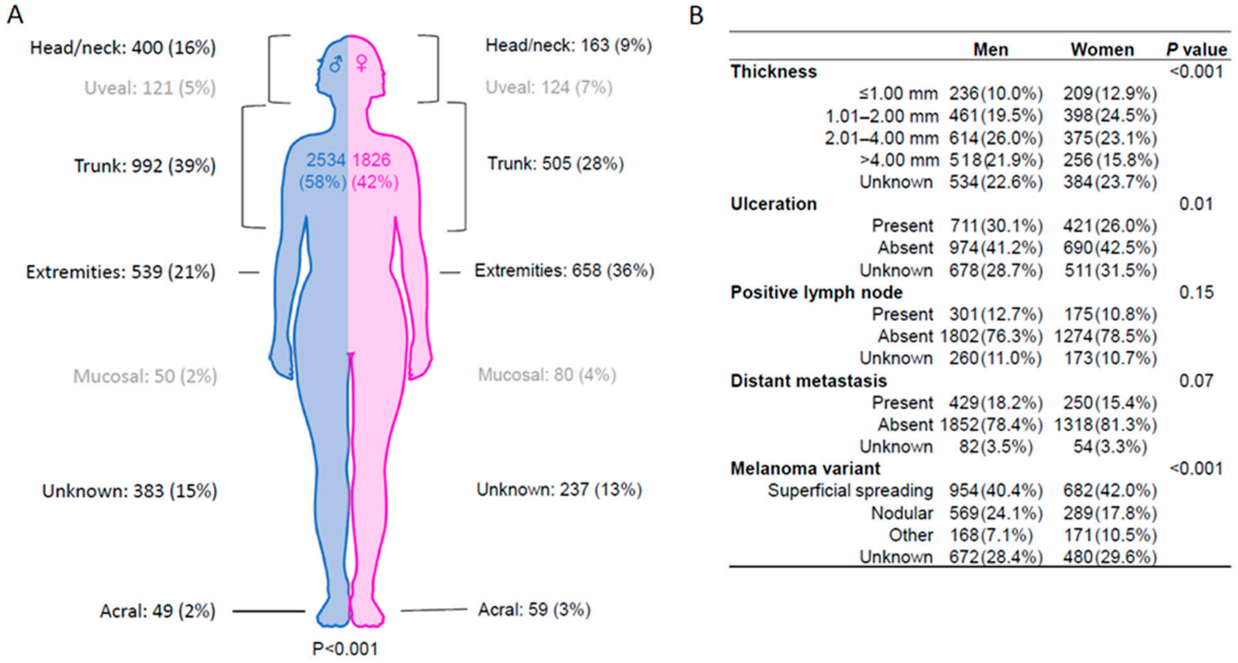

Figure S1. Characteristics of primary tumors of men and women. (A) Anatomical location of the primary tumor. (B) Characteristics of the primary cutaneous melanoma, excluding uveal and mucosal melanoma.

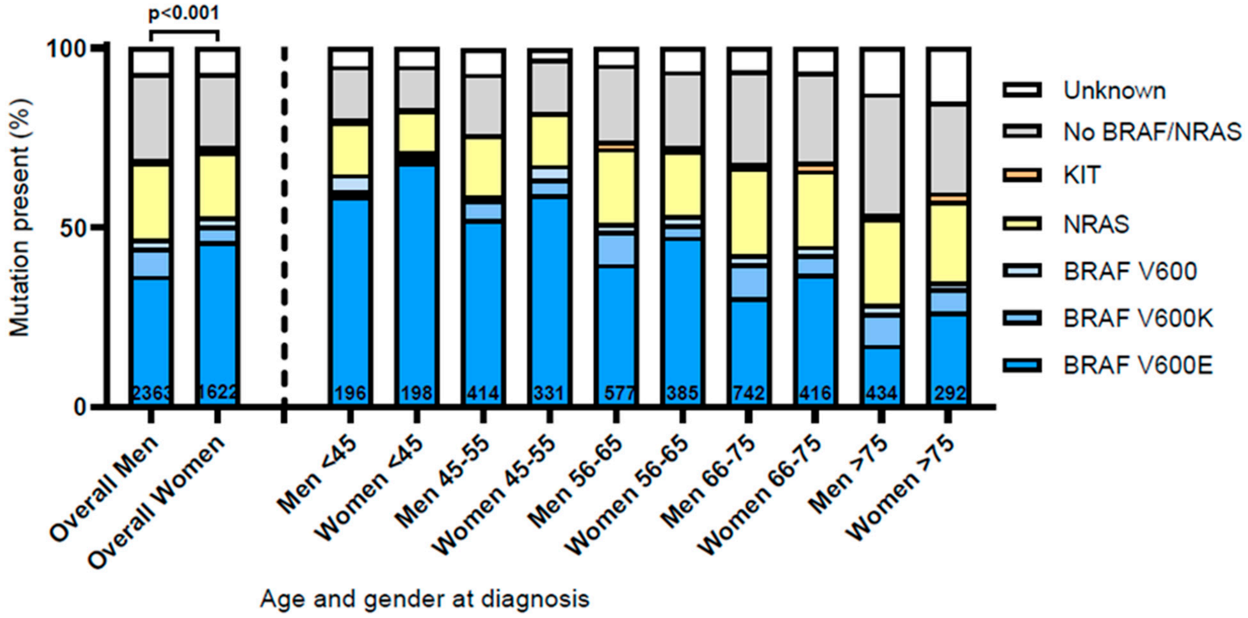

Figure S2. Mutational pattern of the tumor in men and women with advanced melanoma, stratified by age-groups. In the category "BRAF V600" patients with a targetable BRAF V600 mutation are included for whom the BRAF V600 subtype was not registered.

Table S1. Best overall response rate following systemic therapy.

Initial treatment	Men, *N* (%)	Women, *N* (%)	OR (95% CI)	*P* value	adjOR (95%)	*P* value
**BRAF/MEK inhibition**	561	430				

PD	98 (17.5)	65 (15.1)				
SD	169 (30.1)	115 (26.7)				
PR	264 (47.1)	224 (52.1)				
NED/CR	30 (5.3)	26 (6.0)				
ORR	294 (52.4)	250 (58.1)	1.26 (0.98-1.63)	0.07	1.27 (0.99-1.64)	0.06
**Anti-CTLA-4**	253	149				
PD	109 (43.1)	67 (45.0)				
SD	93 (36.8)	55 (36.9)				
PR	31 (12.3)	15 (10.1)				
NED/CR	20 (7.9)	12 (8.1)				
ORR	51 (20.2)	27 (18.1)	0.88 (0.52-1.47)	0.62	0.84 (0.49-1.43)	0.52
**Anti-PD-1**	501	309				
PD	111 (22.2)	94 (30.4)				
SD	124 (24.8)	57 (18.4)				
PR	180 (35.9)	102 (33.0)				
NED/CR	86 (17.2)	56 (18.1)				
ORR	266 (53.1)	158 (51.1)	0.92 (0.70-1.23)	0.59	0.93 (0.70-1.24)	0.62
**Anti-CTLA-4 + anti-PD-1**	107	57				
PD	32 (29.9)	11 (19.3)				
SD	20 (18.7)	8 (14.0)				
PR	46 (43.0)	30 (52.6)				
NED/CR	9 (8.4)	8 (14.0)				
ORR	55 (51.4)	38 (66.7)	1.89 (0.97-3.69)	0.06	2.09 (1.04-4.20)	0.04

Treatment outcome was classified as progressive disease (PD), stable disease (SD), partial response (PR), or complete response (CR). Overall response rate (ORR) is defined as the proportion of patients who have a partial or complete response to therapy. Odds ratios (OR) were calculated using logistic regression and adjusted ORs (adjOR) were adjusted for age, ECOG performance status, LDH level, presence of brain metastases, presence of distant metastasis in ≥3 organ sites.

Table S2. Survival in different age categories.

		**Premenopausal (≤45 years)**				**Menopausal (46-59 years)**				**Postmenopausal (≥60 years)**			
		**Events/total (*N*)**				**Events/total (*N*)**				**Events/total (*N*)**			
		**Men**	**Women**	**HR (95% CI)**	**adjHR (95% CI)**	**Men**	**Women**	**HR (95% CI)**	**adjHR (95% CI)**	**Men**	**Women**	**HR (95% CI)**	**adj HR (95% CI)**
All patients	OS	123/218	109/215	0.85 (0.66-1.10)	0.96 (0.74-1.25)	335/614	262/451	1.08 (0.92-1.27)	1.00 (0.85-1.18)	988/1531	578/956	0.86 (0.78-0.95)	0.87 (0.78-0.96)
	DSS	112/218	87/215	0.75 (0.57-0.99)	0.84 (0.63-1.11)	284/614	208/451	1.01 (0.84-1.21)	0.92 (0.77-1.11)	713/1531	414/956	0.86 (0.76-0.97)	0.88 (0.78-1.00)
	CR	112/218	87/215	0.73 (0.55-0.96)	0.77 (0.58-1.03)	284/614	208/451	0.98 (0.82-1.17)	0.90 (0.74-1.09)	713/1531	414/956	0.90 (0.80-1.01)	0.94 (0.83-1.07)
Initial systemic treatment													
BRAF/MEK inhibition	OS	55/82	50/68	1.22 (0.83-1.80)	1.34 (0.90-1.99)	136/195	122/165	1.20 (0.94-1.53)	1.03 (0.80-1.33)	266/337	156/230	0.68 (0.56-0.83)	0.69 (0.57-0.85)

	DSS	50/82	38/68	1.02 (0.67-1.55)	1.11 (0.72-1.72)	121/195	98/165	1.08 (0.83-1.41)	0.90 (0.67-1.18)	204/337	123/230	0.71 (0.56-0.88)	0.75 (0.59-0.94)
	CR	50/82	38/68	0.92 (0.60-1.39)	0.95 (0.62-1.46)	121/195	98/165	1.00 (0.77-1.31)	0.79 (0.59-1.06)	204/337	123/230	0.81 (0.66-1.02)	0.89 (0.70-1.13)
	PFS	46/82	39/68	1.01 (0.66-1.55)	0.90 (0.55-1.48)	128/195	105/165	1.06 (0.82-1.37)	0.93 (0.71-1.22)	242/337	148/230	0.71 (0.57-0.87)	0.72 (0.58-0.89)
Anti-CTLA-4	OS	22/28	12/22	0.60 (0.30-1.22)		49/76	22/34	0.91 (0.55-1.51)		116/169	68/98	0.93 (0.69-1.25)	1.00 (0.73-1.38)
	DSS	20/28	11/22	0.61 (0.29-1.28)		43/76	16/34	0.74 (0.42-1.32)		90/169	56/98	0.98 (0.71-1.37)	1.05 (0.74-1.49)
	CR	20/28	11/22	0.60 (0.23-1.27)		43/76	16/34	0.68 (0.40-1.17)		90/169	56/98	1.04 (0.75-1.43)	1.05 (0.73-1.49)
	PFS	27/28	20/22	0.97 (0.54-1.73)		67/76	30/34	0.96 (0.63-1.49)		153/169	90/98	0.94 (0.73-1.23)	0.94 (0.71-1.24)
Anti-PD-1	OS	6/29	13/37	1.62 (0.61-4.26)		40/116	34/84	1.38 (0.87-2.18)		164/391	91/215	1.00 (0.77-1.29)	0.97 (0.75-1.27)
	DSS	5/29	10/37	1.48 (0.51-4.34)		32/116	24/84	1.21 (071-2.06)		119/391	72/215	1.09 (0.81-1.46)	1.02 (0.75-1.38)
	CR	5/29	10/37	1.40 (0.50-3.97)		32/116	24/84	1.12 (0.67-1.91)		119/391	72/215	1.11 (0.83-1.49)	1.09 (0.79-1.50)
	PFS	17/29	22/37	1.17 (0.62-2.21)		72/116	51/84	1.13 (0.79-1.63)		244/391	138/215	1.05 (0.85-1.30)	1.02 (0.82-1.26)
Anti-CTLA-4 + anti-PD-1	OS	7/16	5/17	0.70 (0.22-2.21)		15/44	3/23	0.42 (0.12-1.47)		28/60	10/30	0.81 (0.39-1.68)	
	DSS	7/16	4/17	0.56 (0.16-1.91)		14/44	2/23	0.30 (0.07-1.33)		26/60	9/30	0.79 (0.37-1.69)	
	CR	7/16	4/17	0.53 (0.16-1.78)		14/44	2/23	0.30 (0.07-1.31)		26/60	9/30	0.80 (0.37-1.70)	
	PFS	11/16	7/17	0.63 (0.25-1.64)		28/44	8/23	0.52 (0.23-1.14)		38/60	17/30	1.02 (0.56-1.84)	

Adjusted hazard ratios (adj HR) were adjusted for: sex, age, ECOG performance status, LDH, ≥ 3 organ sites affected, the presence of brain metastases, and BRAF V600 mutation status. Only for patients treated with targeted therapy was the BRAF V600 mutational status not included in the Cox proportional hazard model. Events and total number of male and female patients is shown, followed by hazard ratio and corresponding 95% confidence interval, and the adjusted hazard ratio with 95% confidence interval for overall survival (OS), disease specific survival (DSS), and progression free survival (PFS). In the competing risk (CR) model the sub-distribution adjusted hazard ratio is shown.
